# RAS as Supporting Actor in Breast Cancer

**DOI:** 10.3389/fonc.2019.01199

**Published:** 2019-11-12

**Authors:** Mirco Galiè

**Affiliations:** Department of Neuroscience, Biomedicine and Movement, University of Verona, Verona, Italy

**Keywords:** RAS, breast cancer, oncogene, mutations, signal transduction

## Abstract

Oncogenic activation of RAS isoforms leads tumor initiation and progression in many types of cancers and is gaining increasing interest as target for novel therapeutic strategies. In sharp contrast with other types of cancer, the importance of RAS in breast tumorigenesis has long been undermined by the low frequency of its oncogenic mutation in human breast lesions. Nevertheless, a wealth of studies over the last years have revealed how the engagement of RAS function might be mandatory downstream varied oncogenic alterations for the progression, metastatic dissemination, and therapy resistance in breast cancers. We review herein the major studies over the last three decades which have explored the controversial role of RAS proteins and their mutation status in breast tumorigenesis and have contributed to reveal their role as supporting actors, instead of as primary cause, in breast cancer.

## Introduction

RAS G-proteins mediate the signal transduction through the transmembrane receptors. In humans, there are four highly homologous ≈21 KDa RAS isoforms: HRAS, encoded by the *Harvey rat sarcoma viral oncogene homolog (HRAS)*, NRAS, encoded by *neuroblastoma RAS viral (v-ras) oncogene homolog (NRAS)*, and KRAS4A and KRAS4B, alternative splice variants of the *Kristen rat sarcoma viral oncogene homolog (KRAS)*. RAS proteins function as binary switches that cycle between an active (“on”) GDP-bound to an inactive (“off”) GTP-bound state depending on the activation status of the upstream receptors. The switch between “on” and “off” states is modulated by the complementary action of enzymes that promote either the GDP to GTP exchange (guanine exchange factors, GEFs) or the conversion back to GDP-bound form (GTPase-activating proteins, GAPs). The multiplicity of GTPases and GAPs allows the function of RAS to be finely regulated depending on the variety of extracellular and intracellular signal inputs. RAS proteins activate a hierarchical cascade of intersecting pathways which modulate biological functions such as cell proliferation, apoptosis, motility, metabolism, immune evasion. Dysregulation of RAS function is largely associated with tumorigenesis. This may rely either on genomic mutations which alter the RAS-intrinsic structure or on alteration of RAS regulating factors, which enhances RAS expression and activity.

This review is an effort to recapitulate more than 30 years of studies on RAS oncogenes and breast cancer, with the aim to reconcile two apparently conflicting evidences arisen by these studies: (1) experimental studies on cancer cells and murine models have demonstrated that RAS oncogenes and their mutations have a strong potential in breast cancer initiation and progression as it does in other type of cancers; (2) clinical studies have demonstrated that, actually, the incidence of tumorigenic RAS mutations in human breast cancers is marginal, in sharp contrast with other types of cancer.

## Oncogenic Mutations of RAS in Human Cancers

**Figure 1:**
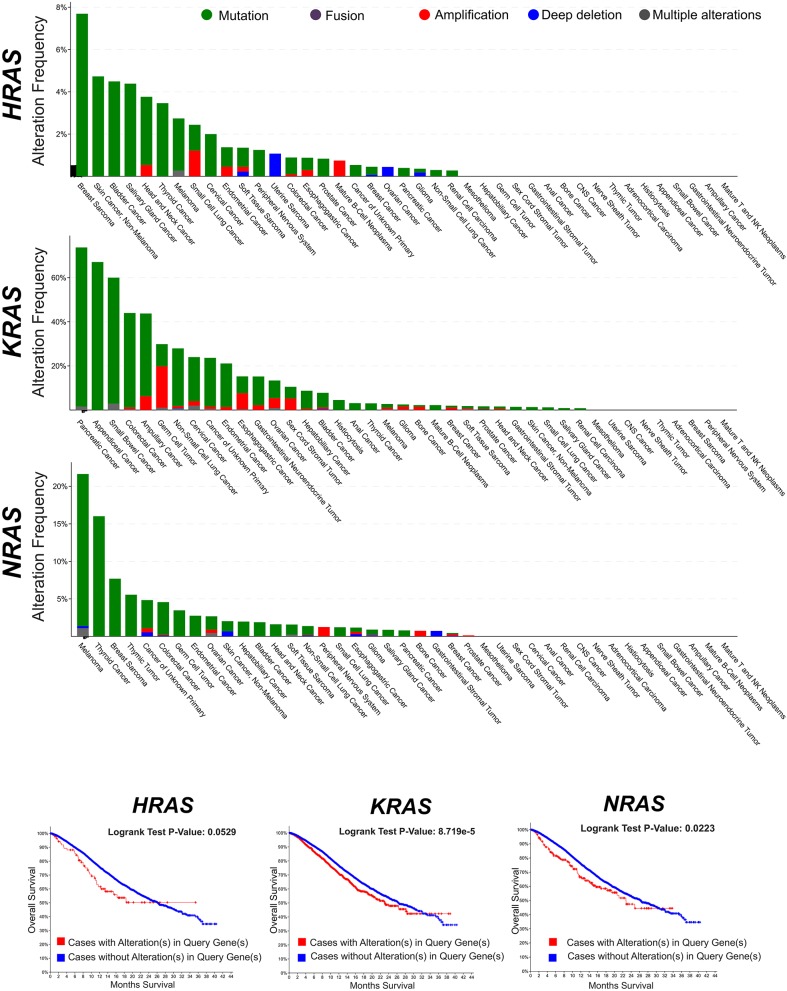
Frequency of genomic alterations (mutation, fusion, amplification, deep deletion, multiple alterations) of the RAS genes (*HRAS, KRAS, NRAS*) across different tumor types of the MSK-IMPACT Clinical Sequencing Cohort (8). Data have been accessed through cBioportal for Cancer Genomics website (https://www.cbioportal.org).

RAS genes were the first mutated genes identified in human cancer (1–3). The discovery in the late 1970s that their gain-of-function mutations were able to trigger tumorigenesis inaugurated the modern molecular oncology and posed the basis of the molecularly targeted anticancer drug discovery (4). To date, hundreds of genes have been identified which harbor oncogenic mutations, but the RAS genes still remain amongst the most frequently mutated oncogene families in cancer. The oncogenic activation of RAS genes is usually caused by a single point mutation (5–7) which impair the RAS responsiveness to the GAP-mediated modulation and locks RAS and their downstream pathways in a persistently active state. Traditional studies (first carried out for the HRAS oncogenic allele) have shown that a single oncogenic RAS gene could transform cells *in vitro* and could provide them with the ability to induce tumors in mice (3). Twenty five percent of human cancers display missense gain-of-function mutations in at least one of the RAS genes and in 98% of the cases mutations are found at one of the mutational hotspots G12, G13, and Q61 (COSMIC v75). Not all RAS isoforms are mutated equally, with KRAS displaying the highest frequency. Also, mutations of specific RAS isoforms exhibit marked preferences for different tumor types and different impact on clinical outcome (Figure 1).

## Oncogenic Activation of RAS in Breast Cancer

Mammary cell lines have served as tumor models for many seminal studies which demonstrated the tumorigenic potential of RAS oncogenes. These studies have shown that oncogenic RAS mutations constitutively enhance mammary cell interaction with basement membrane, alter the tridimensional growth in collagen gel, induce anchorage-independent phenotype, invasiveness, tumorigenic potential, secretion of TGF-β and IGF-1, activation of EGFR, mitogen-activated protein kinase (MAPK), and estrogen-insensitivity (9–18). Single copies of mutant KRAS cooperate with mutant PIK3CA to induce tumor transformation in immortalized human epithelial cells (19). Conditional expression of Ki-RasG12V in the mammary cells induces estrogen receptor alpha (ERα)-positive adenocarcinoma in mice (20), while HRAS Q61 drives breast adenomyoepitheliomas (21).

Several pathways and downstream effectors have been identified which mediate the tumorigenic phenotype induced by oncogenic RAS mutations in mammary cells. Activated NRAS oncogene and its homolog NRAS proto-oncogene act through the same pathway for *in vivo* tumorigenesis (22). Oncogenic RAS mutations support cancer progression and metastatic dissemination through the modulation of the ΔNp63, a amino-terminal truncated isoform of p63, a member of the p53 family of transcription factors (23, 24). Oncogenic RAS mutations promotes TFG-β-induced epithelial-mesenchymal transition through the activation of leukotriene B4 receptor-2-linked cascade (25). Mutated RAS associates with the induction of cyclooxygenase-2 (COX-2) expression in human breast cancer cell lines (26). Activated HRAS induces the invasive phenotype in breast epithelial cell lines through the recruitment of p38 (27, 28). Invasion of breast carcinoma cells also relies on activated Ras-mediated stimulation of E2F and a consequent E2F-mediated modulation of integrin α6β4 (29). Oncogenic RAS mutation regulates the activity of CXCL10 and its receptor splice variant CXCR-B (30). Id1 and activated RAS cooperate to subvert the cellular senescence response and to induce metastatic dissemination in mammary carcinoma (31). Focal adhesion kinase signaling is required for activated RAS and PI3K-dependent breast tumorigenesis in mice and humans (32). Dominant negative Ras activates the Raf-Mek-Erk signal transduction pathway and induces lactogenic hormone-induced differentiation (33). Activated RAS signals centrosome amplification through cyclin D1/Cdk4 and Nek2 (34). Autophagy is critically implicated in malignant transformation by oncogenic KRAS mutations and is promoted by the reactive-oxygen species-mediated JNK activation through up-regulation of ATG5 and ATG7 (35). RAS oncogenesis is accelerated by p21WAF1/CIP1 depletion in mammary cancer (36), while p21CIP attenuates RAS- and c-MYC-dependent epithelial-to-mesenchymal transition and cancer stem cell-like transcriptional profile *in vivo* (37). Gadd45a induces apoptosis and senescence in Ras-driven mammary cancers through activation of c-jun NH2-terminal kinase and p38 stress signaling (38). HMGA1a regulates genes involved in the RAS/ERK mitogenic signaling pathway, including KIT ligand and caveolin 1 and 2 (39). Oncogenic RAS mutations induce metabolic rearrangement in breast cancer as part of their tumorigenic program. Activated c-ha-Ras induces loss of fatty-acid delta desaturating ability in human mammary epithelial cells (40). Moderate restriction of energy intake hampers v-Ha-ras-induced mammary tumorigenesis (41). PI3K and KRAS cooperate to stimulate *de novo* lipid synthesis through mTORC1 and SREBP (42).

## RAS Hyperfunction in Breast Cancer

**Figure 2:**
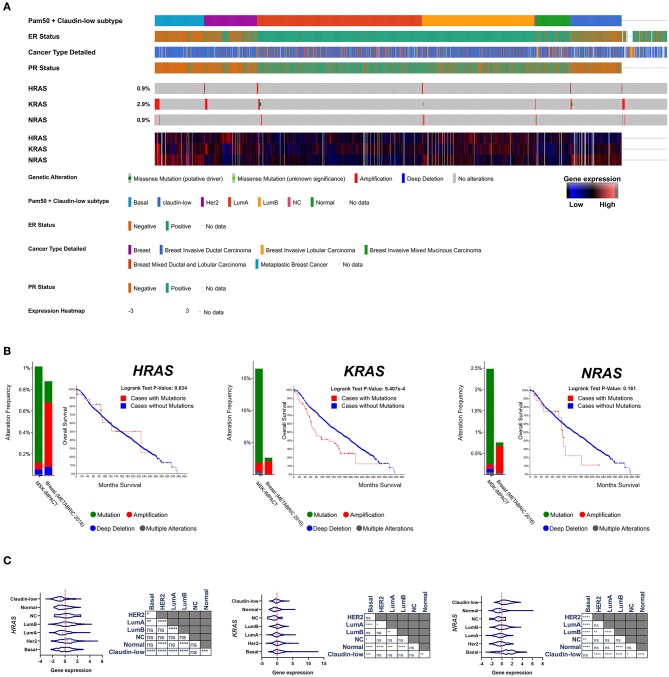
**(A)** Frequency of genomic alterations and heatmap of gene expression of the RAS genes (*HRAS, KRAS, NRAS*) across 2,509 breast tumor samples of the METABRIC cohort (48), assigned to the major intrinsic subtypes (on the basis of the PAM50 profile). **(B)** The fraction of breast tumors (cases) with genomic alterations (METABRIC cohort, 2,509 breast tumors) of the RAS genes are reported in comparison with the fraction of tumor with genomic alteration in a pan-tumor cohort (MSK-IMPACT, 10945). Kaplan-Mayer curve are reported for each RAS isoform comparing the overall survival of the breast tumors with or without genomic alterations (METABRIC cohort, 2,509 breast tumors). **(C)** Gene expression level of RAS isoforms across the different subtypes of breast cancer are compared and statistically evaluated by ANOVA *t*-test (Turkey *post-hoc*) ^*^p < 0.05; ^**^p < 0.01; ^***^p < 0.001; ^****^p < 0.0001. Data have been accessed through cBioportal for Cancer Genomics website (https://www.cbioportal.org).

After the first identification of the tumorigenic potential of oncogenic RAS mutations *in vitro*, a great effort has been made in search for RAS mutations in human cancers, and their role in driving tumorigenesis (43). The most remarkable finding was the discovery of the stricking incidence of oncogenic KRAS mutations in colon (44, 45), lung (46), and pancreatic carcinomas (47) (Figure 1). According to what found in other tumor types, KRAS confirms to be the most frequently mutated RAS isoform in breast cancer (Figure 2A) and its mutation, unlike mutations of HRAS and NRAS, is strongly associated with the poor clinical outcome (Figure 2B). Nevertheless, the frequency of RAS mutations in human breast cancer proven to be much lower than expected (49) (Figures 1, 2). This stands against a critical role of RAS oncogenic activation as the primary driver of the breast cancer initiation and progression in humans and has discouraged for many years the effort to investigate RAS proteins as potential targets for breast cancer therapies (Figure 2A). Also, human RAS oncogene, unlike their retroviral counterpart, cannot transform primary cells without the cooperation by a second oncogene such as MYC or adenovirus E1A.

However, the oncogenic function of RAS proteins does not rely completely on gene mutations. RAS protein overexpression, hyperactivation of upstream RAS activators, such as receptor tyrosine kinases, perturbation in the activity of RAS regulators, such as GEFs and GAPS, all may contribute to promote and sustain tumorigenicity (50, 51).

### RAS Hyperfunction Induced by Upstream Tumorigenic Effectors

There are a wealth of evidence that stratified over the last 3 decades which have established a role of RAS as supporting actor in breast cancer downstream the dysregulated action of oncogenic pathways and effectors. RAS proteins serve as hub of the major intracellular signaling pathways which govern cell growth, motility, angiogenesis, immune escape. Hence, it is quite clear that the engagement of RAS function is mandatory for many oncogenic factors to be able to propagate their signals and execute their aberrant programs, while its inhibition may dampen upstream tumorigenic signals. Studies in the early 1990s reported that in 71% of human breast cancers the expression of RAS proteins was higher than in normal breast tissues and correlated with that of p185/HER-2. Interestingly, NRAS and HRAS result to be overexpressed in basal-like and HER2 tumors, the most aggressive subtypes of breast cancer (52, 53) (Figure 2A). HER2, as well as its cognate epidermal growth factor receptor (EGFR), is coupled to the Ras signaling by interaction with the adaptor protein Grb2, and Sos, a Ras GDP-GTP exchange factor. The overexpression of these receptors in breast cancer cells amplifies the RAS signaling pathway (54). Consistently, the tyrosine kinase inhibitors have been shown to hamper breast cancer cell proliferation at least in part by the inhibition of signal transduction processes potentially mediated through RAS (55). RAS overexpression associates with p53 loss, HER2 amplification/overexpression and aneuploidy in infiltrating ductal carcinomas (56). RAS is required also for the mammary tumorigenesis induced by the oncogene MYC, although in an inducible mouse model of c-MYC-driven mammary tumorigenesis the spontaneous occurrence of secondary RAS mutations was necessary to prevent the full regression of tumors upon c-MYC deinduction (57). Pin1, a prolyl isomerase which regulates the conformation of a subset of phosphorylated Ser/Thr-Pro motifs, is overexpressed in human tumors and interacts with Ras signaling in increasing c-Jun transcriptional activity toward cyclin D1 (58). Breast cancer displays the downregulation of the RAS/MAPK inhibitor proteins sprouty 1 and 2 (59). RAS functions downstream Rab-coupling protein RCP (also known as RAB11FIP1), a breast cancer-related oncogene (60). Bone Morphogenetic Protein 1 (BMP1) cooperates with HRAS to induce metastatic breast cancer (61). RAS signaling amplification has been reported to play a crucial role in metastatic progression and poor clinical outcome of luminal breast cancer patients (62). MicroRNA-382-5p accelerates breast cancer progression by modulating the RERG/RAS/ERK signaling axis (63). Pharmacological inhibition of SHP2 phosphatase has been recently shown to reduce the proliferation rate of receptor-tyrosine-kinase-driven human cancer cells *in vitro* and *in vivo* through the inhibition of the RAS-MAPK signaling (64). BCL-XL directly modulates RAS signaling to favor cancer cell stem-like phenotype (65).

### RAS Hyperfunction Induced by Altered Activity of RAS Regulators

RAS hyperfunction with tumorigenic effects can be induced by the altered activity of RAS-specific regulators. R-RasGTPase activating protein mediates the interaction between estrogen and insulin signaling pathways in breast cancer cells (66) and affects the motile phenotype of breast epithelial cells through the modulation of Rho/Rho-kinase (67). On the other hand, RAS-GTPase inhibition promotes apoptosis in tumor cells (68). The RasGAP gene, RASAL2, functions as a tumor and metastasis suppressor in human luminal breast cancer (69) but promote triple-negative breast cancer progression through RAC1 activation (70). The Rho GTPase Rnd1 dampens mammary tumor progression and EMT by restraining RAS-MAPK signaling (71, 72). R-Ras2, a transforming GTPase that shares downstream effector with Ras proteins, promotes tumor progression in a PI3K-dependent and signaling autonomous manner although its prometastatic role requires other priming oncogenic signals and downstream effectors (73). Transposon insertion in one of two RASGAP genes, neurofibromin1 (Nf1) and RAS p21 protein activator (Rasa1), might function as the causal role of the mammary tumor development in a tumor mouse model generated by the activation of a mutagenic T2Onc2 transposon via expression of a transposase driven by the keratin K5 promoter in a p53^+/−^ background (74).

## RAS in Triple-Negative/Basal-Like Breast Cancer

Triple negative breast cancer (TNBC) is a heterogeneous group of tumors defined on the basis of their negativity for Estrogen receptor, Progesterone Receptor and HER2. They account for ≈15% of breast tumors and are statistically associated to poor prognosis. TNBC phenotype and clinical outcome partially overlap those of the basal-like breast cancer subtype previously identified on the basis of the gene expression profiling (52, 53), although the identification between these two categories of breast tumors is controversial (75). RAS activity and its regulators have been reported to play a role in the progression of TNBC/basal-like tumors. A 3′-untranslated region of KRAS variant has been identified which regulates the development of TNBC (76). KRAS(G12D) provides human mammary basal cells and luminal progenitors with the ability to produce serially transplantable, polyclonal, invasive ductal carcinomas into immunodeficient mice, which display a dramatic clonal diversification (77). miR-143/145 loss-of-function amplifies the tumorigenic potential of PTEN-deficient basal-like breast tumor cells at least partially through the induction of RAS signaling. In humans, miR-145 deficiency correlates with enhanced RAS-pathway activity in basal-like breast cancer, and patient with combined PTEN/miR-145 loss or PTEN-loss/high RAS-pathway activity exhibit poor clinical outcome (78). Also, wild-type NRAS, upregulated in basal-like breast cancer (Figure 2A), promotes tumorigenesis through IL-8 secretion via JAK2 activation (79). RAS-MAPK pathway activation promotes immune-evasion in triple negative breast cancer (80). High level of ERK1/2 phosphorylation, a readout of Ras signaling activation, has been found in metastatic sites relative to primary breast tumors and is more common in TNBC/basal-like cancers (81). Transcriptional signature of RAS/MAPK pathway activation is highly prevalent in TNBC/basal-like cancers compared to other subtypes of breast cancer (82, 83).

## RAS in Breast Cancer Therapy

Other than being a potent mediator of tumor transformation and progression, RAS might also confer resistance to therapies in breast cancer (84). RAS induces resistance to Cis-platinum by increasing GST-pi expression (85) and ERCC1 (86). Oncogenic RAS mutations cause resistance to the growth inhibitor insulin-like growth factor binding protein-3 (IGFBP-3) (87). Also, RAS induces resistance to lapatinib which might be overcome by MEK inhibition (88). RAS/Raf-1/MAPK pathway affects response to tamoxifen but not chemotherapy in breast cancer patients (89). Raf-1 functions as an effector of RAS in the radiation-response (90).

The role of RAS in breast tumorigenesis and resistance to therapies provides the rationale to assess RAS as target in breast cancer treatment. Three decades of studies contributed to rise the notion that RAS oncogenes are “undruggable,” due to its conformational architecture, which lacks of pockets to facilitate the binding of small inhibitors, and its picomolar affinity for the nucleotide substrate. However, recent technologies and approaches have renewed the challenge to thwart cancer by targeting RAS directly or through its downstream signaling pathways. Direct approaches currently under investigation are addressed to enhance GTP hydrolytic activity of RAS, to inhibit its nucleotide exchange function or to prevent its interaction with downstream effectors (91, 92). These approaches are providing encouraging results at preclinical stages, but none of them have entered clinical practice thus far.

A reliable alternative approach consists in blocking the RAS downstream pathways (93). As for breast cancer, it holds great promise the therapeutic use of inhibitors of the Ras/MAPK pathway. FDA-approved Inhibitors of MEK, a central node in the Ras/MAPK pathway, specifically inhibit proliferation of TNBC/Basal-like cancer cell lines (83) and may complement chemotherapeutic treatments in xenograft models (82). MEK inhibition has been shown to prevent epithelial-mesenchymal transition and metastatic potential of tumor cells by targeting cancer stem cell compartment (94). Although the phase I studies have shown a scarce efficacy of MEK in humans, the combination with neoadjuvant or post-operative treatments might represents a promising alternative (95, 96).

## Concluding Remarks

Decades of studies have contributed to unveil the primary role of RAS oncogenes in leading tumor initiation in many types of cancers. For reasons that are still unknown, breast cancer is not amongst them. Although oncogenic RAS is able to transform mammary cancer cell lines *in vitro*, the marginal incidence of RAS mutations in clinics does not support a primary role of RAS proteins in breast tumor etiology. Nevertheless, a wealth of studies over many years have demonstrated the importance of RAS function in the progression, metastatic dissemination and therapy resistance in breast cancers, regardless the molecular trigger they are initiated by, thus contributing to draw for RAS proteins a crucial role as supporting actors in breast tumorigenesis.

## Author Contributions

MG: conceptualization, writing, and financial support.

### Conflict of Interest

The author declares that the research was conducted in the absence of any commercial or financial relationships that could be construed as a potential conflict of interest.
